# Chloroplast Phylogenomics and Evolutionary History of the Alpine Endemic *Eutrema scapiflorum*

**DOI:** 10.3390/ijms27125195

**Published:** 2026-06-08

**Authors:** Ting Lv, Xiayu Hu, Lizhi Guo, Jiasheng Ju, Yu Zhang, Nan Tang

**Affiliations:** 1 College of Agriculture and Animal Husbandry, Qinghai University, Xining 810016, China; lvtingiu@163.com; 2 Key Laboratory of Qinghai Province for Landscape Plants Research, Plaeau Flower Research Centre, Qinghai University, Xining 810016, China; 3 College of Agronomy, Northwest A&F University, Yangling 712100, China; huxiayu522@163.com (X.H.); yuzhang98@nwafu.edu.cn (Y.Z.); 4 Qinghai Institute for Drug Control, Xining 810016, China; paul_567@163.com; 5 Food and Drug Testing Center, Xianyang 712000, China; starjjs0616@163.com

**Keywords:** *Eutrema scapiflorum*, chloroplast genome, high-altitude adaptation, middle Miocene, phylogenomics

## Abstract

In this study, we sequenced, assembled, and characterized the first complete chloroplast (cp) genome of *Eutrema scapiflorum*, an alpine species endemic to the Qinghai–Tibet Plateau (QTP). The assembled plastome is 153,041 bp in length and exhibits a typical quadripartite structure, comprising a large single-copy (LSC) region of 83,547 bp and a small single-copy (SSC) region of 17,506 bp, which are separated by two inverted repeats (IRs) of 25,994 bp each. Structurally, the genome encodes 132 unique genes, including 87 protein-coding genes, 37 tRNA genes, and 8 rRNA genes. Comparative analysis across eight species revealed that genome size variation is primarily driven by the SSC region. Notably, the IR/SC boundaries in *E. scapiflorum* are highly conserved, which contrasts with the significant IR expansion observed in *Capsella tenella*. Furthermore, simple sequence repeat (SSR) analysis identified 78 loci, predominantly mononucleotide A/T repeats located in intergenic spacers. Nucleotide diversity analysis pinpointed *accD* and *ycf1* as the most variable genes. Selection pressure analysis indicated that most genes are under purifying selection, while seven protein-coding genes (*ycf2*, *nadhE*, *cemA*, *clpP*, *psbH*, *ycf4*, *nadhB*) exhibited signatures of positive selection (*K*a/*K*s > 1). Subsequently, phylogenomic analyses robustly resolved *E. scapiflorum* within the tribe Arabideae, showing its closest relationship to *Alliaria petiolata*. Divergence time estimation dated the split between *E. scapiflorum* and its closest relative to the middle Miocene (~17.57 Ma). Collectively, these findings provide crucial genomic resources and new insights into the structural evolution, phylogenetic placement, and potential adaptive mechanisms of this alpine species within the Brassicaceae family.

## 1. Introduction

The Brassicaceae (Cruciferae) family represents a major model system in plant biology, providing unparalleled insights into angiosperm evolution, diversification, and adaptation. The family’s ecological amplitude, spanning from tropical lowlands to alpine zones, makes it an ideal group for studying how plants evolve in response to extreme environmental stressors, including high-altitude conditions such as hypoxia, intense ultraviolet radiation, and temperature fluctuations [1]. Among these diverse lineages, the genus *Eutrema* (encompassing taxa formerly circumscribed as *Pegaeophyton*) comprises multiple high-altitude specialists that serve as exemplary models for investigating alpine adaptation [2]. *Eutrema scapiflorum* (Hook.f. & Thomson) Al-Shehbaz & Warwick (syn. *Pegaeophyton scapiflorum*), a representative taxon endemic to the Qinghai–Tibet Plateau and adjacent Himalayas, predominantly inhabits scree slopes and alpine meadows within an elevational range of 3500 to 5400 m [3,4,5]. Beyond its significance in evolutionary ecology, *E. scapiflorum* possesses substantial ethnobotanical value, with traditional applications in treating febrile diseases, trauma, and respiratory or digestive disorders. Recent phytochemical studies have further identified a suite of bioactive secondary metabolites including flavonoids, alkaloids, and glucosinolates that likely contribute to its documented anti-inflammatory, antioxidant, and antimicrobial properties [6,7].

Despite the burgeoning interest in high-altitude adaptation and the widespread application of phylogenomic approaches in Brassicaceae systematics, the complete chloroplast genome of *E. scapiflorum* remains uncharacterized [8,9]. This lack of genomic data hinders the precise resolution of its phylogenetic placement and the investigation of potential plastid-mediated adaptations to extreme environments [10]. Chloroplast genomes are valuable in evolutionary studies due to their generally conserved structure, moderate nucleotide substitution rates, and uniparental inheritance, which facilitate comparative genomic analyses and the detection of signatures of selection [11,12]. In high-altitude plants, chloroplast genes involved in photosynthesis, stress responses, and redox homeostasis may be targets of adaptive evolution, making whole-plastome sequencing a powerful tool for uncovering genomic correlates of alpine colonization [13,14].

Previous molecular phylogenetic studies of short-lived Brassicaceae taxa—often based on limited datasets such as nuclear internal transcribed spacer (ITS) and chloroplast loci like *trnL-F*—have revealed complex patterns of relationships among ephemeral and perennial lineages [15,16]. These analyses suggest that short-lived taxa are phylogenetically scattered rather than forming a distinct clade, pointing to possible convergent evolution of life-history traits and/or incomplete phylogenetic resolution due to limited informative characters [9,17]. To clarify the evolutionary history of *E. scapiflorum* and its relatives, a genome-scale approach is needed—one that integrates structural, comparative, and selection analyses to generate robust phylogenetic hypotheses and to identify genomic features associated with alpine life.

In this study, we present the first complete chloroplast genome assembly of *E. scapiflorum* and employ a comprehensive comparative framework to address the following objectives: (1) characterize the structural features, gene content, repetitive elements, and nucleotide diversity of the plastome; (2) examine IR boundary dynamics, sequence divergence, and substitution patterns across related Brassicaceae species; (3) detect signatures of selection in protein-coding genes that may underlie adaptation to high-altitude environments; and (4) reconstruct a robust phylogeny of Brassicaceae using whole chloroplast genomes to clarify the systematic position of *E. scapiflorum* and estimate divergence times within the family.

Our results not only provide a valuable genomic resource for future studies on alpine adaptation and species delimitation in Brassicaceae, but also contribute to a broader understanding of how chloroplast genome evolution may reflect environmental constraints in one of the world’s most extreme botanical hotspots.

## 2. Results

### 2.1. Structural Characteristics of the Chloroplast Genome

**Figure 1:**
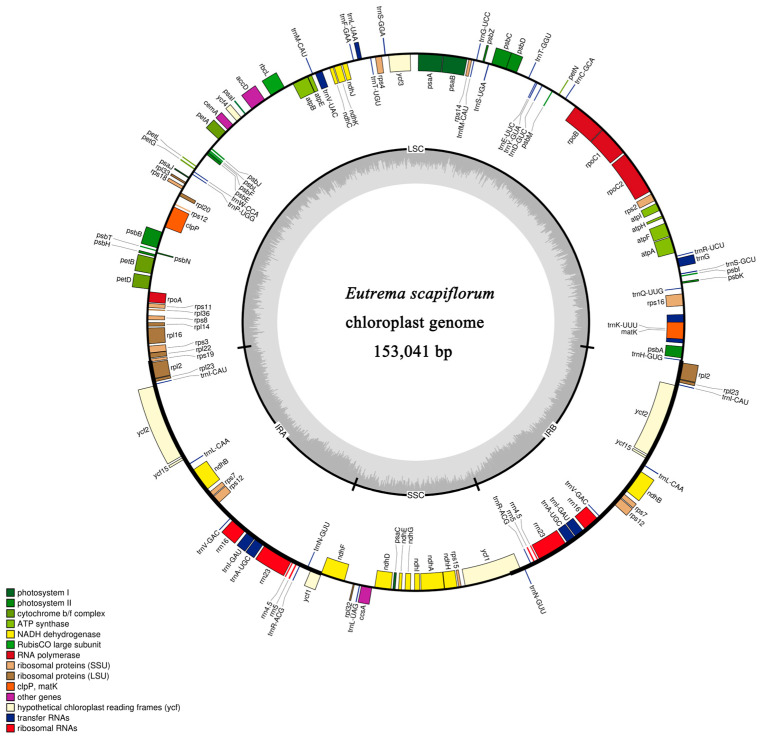
Circularized map of the chloroplast genome of *E. scapiflorum*. The genome shows a typical quadripartite structure: large single-copy (LSC), small single-copy (SSC), and two inverted repeat (IRa and IRb) regions. Genes inside the circle are transcribed clockwise; genes outside are transcribed counterclockwise. Different functional groups are labeled with distinct colors. The inner circle shows the GC content variation across the genome.

The complete chloroplast genome of *E. scapiflorum* is 153,041 bp in length and exhibits a typical quadripartite structure, comprising a large single-copy (LSC) region of 83,547 bp, a small single-copy (SSC) region of 17,506 bp, and a pair of inverted repeat (IR) regions of 25,994 bp each (Figure 1). The overall GC content of the genome is 36.41%, while the GC contents of the LSC, SSC, and IR regions are 34.10%, 29.49%, and 42.44%, respectively. A total of 132 genes were annotated, including 87 protein-coding genes, 37 transfer RNA (tRNA) genes, and 8 ribosomal RNA (rRNA) genes. Among these, 18 genes are duplicated (Table 1). Additionally, introns were identified in 23 genes, of which 19 harbor a single intron and 4 contain two introns (Table 1).

### 2.2. Analysis of SSR Loci

**Figure 2:**
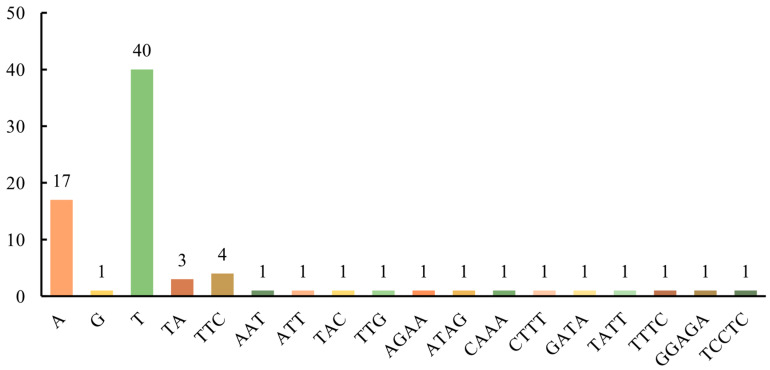
Types and numbers of SSRs in the chloroplast genome of *E. scapiflorum*. Monomers, dimers, trimers, tetramers, and pentamers are shown. No hexanucleotide repeats were detected. Frequencies of each repeat motif are displayed as bar heights.

Simple sequence repeats (SSRs) are tandemly repeated sequences composed of 1–6 nucleotide motifs. A total of 78 SSR loci were identified in the *E. scapiflorum* chloroplast genome. These comprised 58 mononucleotide repeats (A, G, and T), 3 dinucleotide repeats (TA), 8 trinucleotide repeats (TTC, AAT, ATT, TAC, and TTG), 7 tetranucleotide repeats (AGAA, ATAG, CAAA, CTTT, GATA, TATT, and TTTC), and 2 pentanucleotide repeats (GGAGA, TCCTC). No hexanucleotide repeats were detected (Figure 2). Analysis of genomic distribution revealed that 54.30% of the SSR loci were situated in intergenic spacers (IGSs), 32.78% in exons, and 12.91% in introns (Table 2). The high abundance of A/T mononucleotide repeats is a conserved feature consistent with most angiosperm plastomes.

A total of 33 dispersed repeats were detected in the *E. scapiflorum* plastome. These included 17 palindromic, 14 forward, 1 reverse, and 1 complementary repeat (Table 3). Palindromic and forward repeats were the predominant types. The lengths of these dispersed repeats ranged from 30 to 65 bp. The predominance of palindromic and forward repeats aligns with expected patterns in related Brassicaceae species.

### 2.3. Genome Collinearity Analysis

**Figure 3:**
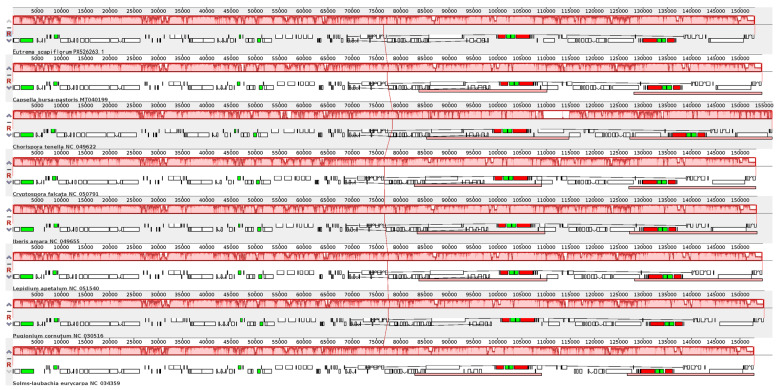
Analysis of chloroplast sequence homology. Locally Collinear Blocks (LCBs) are indicated by colored blocks. Colored blocks represent locally collinear blocks (LCBs) shared among genomes. Blocks above the central are in the forward orientation, while blocks below indicate inversions. Red vertical lines mark LCB boundaries and potential structural variation sites. No large-scale rearrangements were detected among the compared species. All blocks are oriented in the same direction, indicating no large-scale inversions or genome rearrangements. *E. scapiflorum* was used as the reference sequence.

Collinearity analysis was performed using the Mauve model to identify potential genome rearrangements and inversions across the chloroplast genomes of eight species. As shown in Figure 3, the alignment revealed distinct locally collinear blocks (LCBs). All LCBs were oriented in the same direction, with no regions appearing below the central axis, indicating a lack of inversions. Consequently, no rearrangements or inversions were observed, suggesting that the chloroplast genome structure is highly conserved within *E. scapiflorum*.

### 2.4. Analysis of IR Boundary Regions

**Figure 4:**
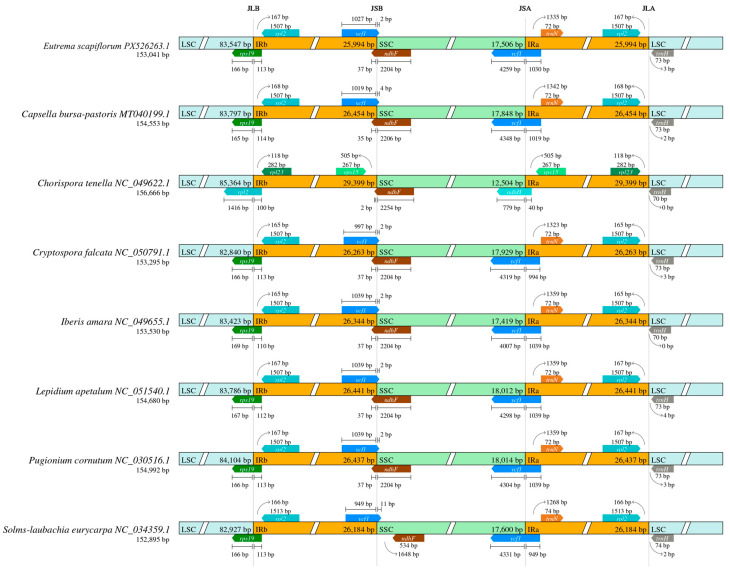
Schematic diagram of chloroplast genome boundary regions of 8 species. JLB: IRb/LSC boundary; JSB: IRb/SSC boundary; JSA: SSC/IRa boundary; JLA: IRa/LSC boundary. Lengths of LSC, SSC, and IR regions are labeled in base pairs (bp). Chorispora tenella shows obvious IR expansion compared with the other species.

To investigate the expansion and contraction of the inverted repeat (IR) regions in chloroplast genomes for *E. scapiflorum*, a comparative analysis of IR boundaries was performed on the chloroplast genomes of eight species. Exact boundary locations and region lengths are summarized in Table 4. The results revealed variation in both total genome size and the lengths of individual regions among the analyzed species (Figure 4). Total genome lengths ranged from 152,895 to 156,666 bp. The lengths of the LSC, SSC, and IR regions ranged from 82,927 to 85,364 bp, 12,504 to 18,014 bp, and 25,994 to 29,399 bp, respectively. Notably, *Chorispora tenella* exhibited significant structural divergence. When *C. tenella* was excluded, the length variation decreased substantially: the LSC range narrowed to 82,927–84,104 bp, the SSC to 17,419–18,014 bp, and the IR to 25,994–26,454 bp. Thus, the SSC region was identified as the primary source of genome size variation. In contrast, *E. scapiflorum* showed a highly conserved structure, with regional lengths consistent with those of the other related species.

### 2.5. Polymorphic Site Analysis

**Figure 5:**
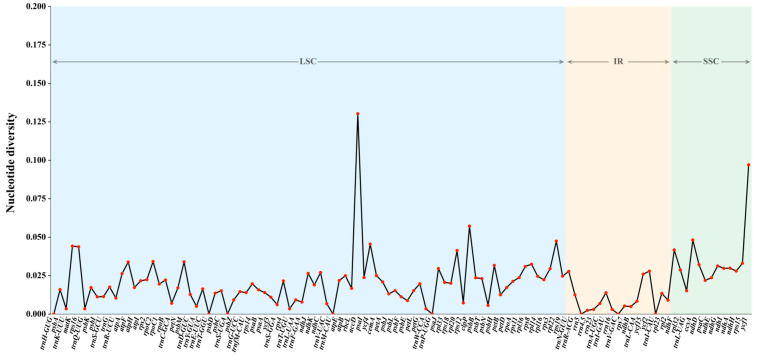
Pi values of chloroplast genome nucleotide polymorphisms in *E. scapiflorum*. Pi values were calculated using DnaSP with a 200-bp window and 600-bp step size. LSC: large single-copy region; IR: inverted repeat region; SSC: small single-copy region. Genes with the highest Pi values are indicated.

Single nucleotide polymorphisms (SNPs) quantify the extent of nucleic acid sequence variation among species. In this study, nucleotide diversity (Pi) across the *E. scapiflorum* chloroplast genome ranged from 0 to 0.13031. Notably, *accD* and *ycf1* were identified as highly variable hotspots, exhibiting exceptionally high Pi values of 0.13031 and 0.09711, respectively (Table S1, Figure 5). To rule out potential artifacts, these high Pi values were manually verified to ensure they were not driven by poor alignment quality, structural variations, or annotation discrepancies. Given their substantial divergence (Pi > 0.09), the observed variations in *accD* and *ycf1* provide sufficient phylogenetic signal, indicating that they can serve as practical and robust molecular markers for future species delimitation and population genetic studies in Eutrema, rather than merely representing descriptive divergent regions.

### 2.6. Ka/Ks Analysis

**Figure 6:**
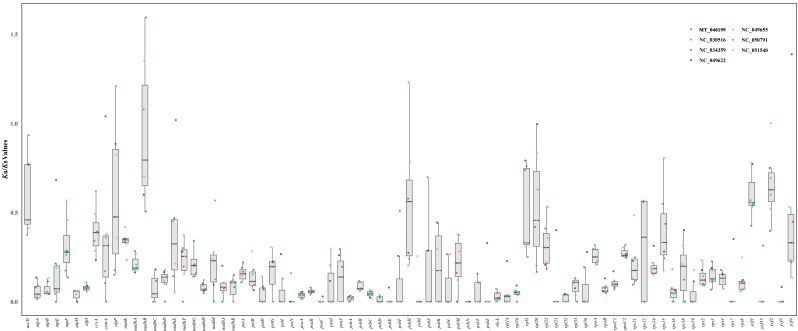
Selection pressure analysis of protein-coding genes in chloroplast genomes of *E. scapiflorum* and eight species. Genes with *Ka*/*Ks* > 1 are marked as candidates for potential positive selection. Most genes show *Ka*/*Ks* < 1, indicating purifying selection.

Selective pressure analysis was performed on 76 protein-coding genes shared among *E. scapiflorum* and seven related species, following the removal of invalid data (*K*a/*K*s = 0 or NA). Seven genes exhibited *K*a/*K*s ratios exceeding 1, including *ycf2*, *ndhE*, *cemA*, *clpP*, *psbH*, *ycf*4, and *ndhB*, with values ranging from 1.001 to 1.594. These ratios indicate that these genes are subject to positive selection. In contrast, the remaining genes showed *K*a/*K*s ratios below 1, suggesting a predominance of synonymous substitutions and the influence of purifying selection (Figure 6).

### 2.7. Phylogenetic and Divergence Time Analysis

**Figure 7:**
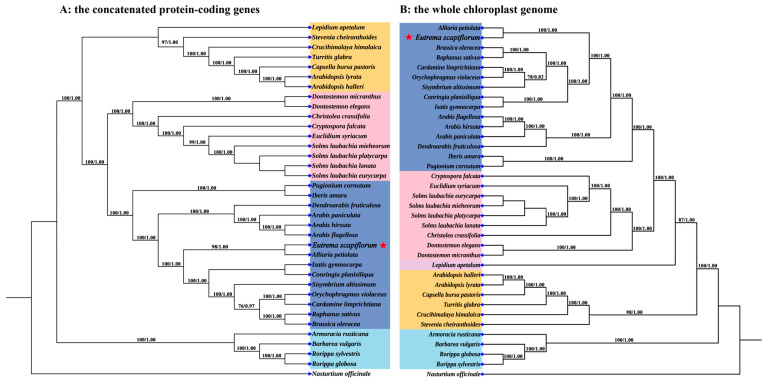
Phylogenetic relationships of 36 Brassicaceae species. The focal species, *E. scapiflorum*, is highlighted with a star (★). (**A**) is the phylogenetic tree constructed by protein-coding sequences, and (**B**) is the phylogenetic tree constructed by chloroplast whole genome sequences. Both phylogenies were reconstructed using Maximum Likelihood (ML) and Bayesian Inference (BI) methods. Numbers at the nodes represent ML bootstrap support values and BI posterior probabilities (ML/BI), respectively. Major clades are consistently color-coded across both panels to facilitate visual comparison.

To elucidate the phylogenetic relationships within *E. scapiflorum*, phylogenetic trees were reconstructed for 36 Brassicaceae species using both complete chloroplast genomes and CDSs. The phylogenetic analysis based on CDSs resolved the species into four highly supported clades (BS = 100, PP = 1.00). Within this topology, *E. scapiflorum* clustered within a stable clade (BS = 100, PP = 1.00) together with *A. petiolata*, and *S. altissimum* from the tribe Sisymbrieae; *A. flagellosa*, *A. hirsuta*, *A. paniculata*, and *D. fruticulosa* from the tribe Arabideae; *B. oleracea*, *C. limprichtiana*, *O. violaceus*, and *R. sativus* from the tribe Brassiceae; *C. planisiliqua* from the tribe Conringieae; and *I. amara*, *I. gymnocarpa*, and *P. cornutum* from the tribe Lepidieae. *E. scapiflorum* was found to be most closely related to *A. petiolata* (Figure 7). In comparison, the tree based on complete chloroplast genomes yielded a similar topology but identified five clades, with *L. apetalum* forming a separate lineage (BS = 87, PP = 1.00). Despite this minor discrepancy, both datasets consistently supported a sister relationship between *E. scapiflorum* and *A. petiolata* (BS = 98/100, PP = 1.00; Figure 7). Overall, the chloroplast genome data provided strong resolution, establishing a reliable phylogenetic framework for *E. scapiflorum*.

**Figure 8:**
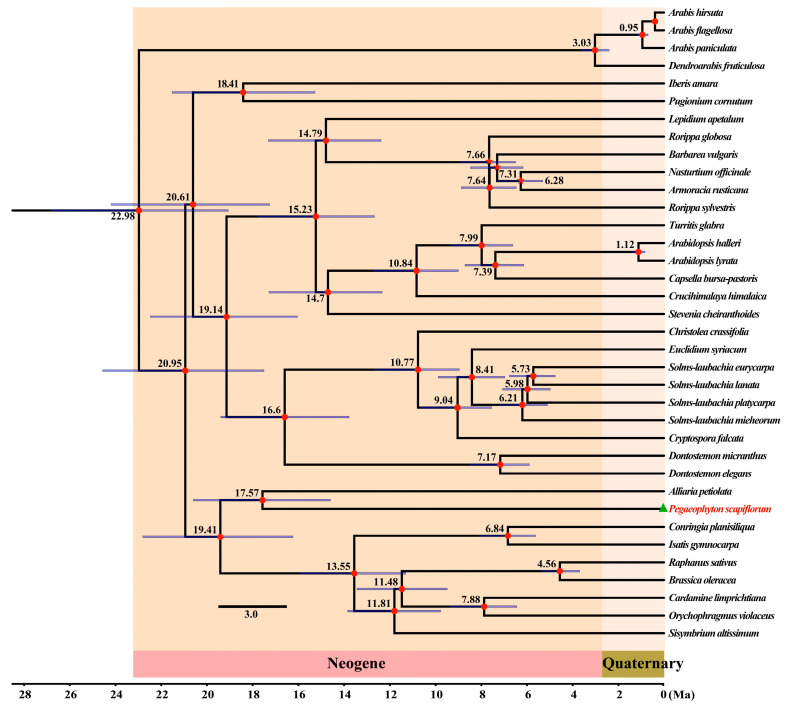
Divergence time estimation based on the phylogenetic tree of chloroplast genomes of 36 species. Numbers at the nodes indicate the median estimated divergence times (Ma). Blue bars represent the 95% highest posterior density (HPD) intervals. The bottom axis displays the geological timescale. The two red stars indicate the calibration points used for divergence time estimation, while the different colors represent distinct geological ages.

Divergence times for *E. scapiflorum* were estimated using a Bayesian relaxed clock model based on CDSs and secondary calibration points. The resulting time-calibrated phylogeny indicates that the analyzed Brassicaceae lineage diversified during the early Miocene, approximately 22.98 Ma (95% highest posterior density: 17.50–24.56 Ma; Figure 8). Subsequently, the divergence between *E. scapiflorum* and *A. petiolata* was estimated to have occurred around 17.57 Ma (95% HPD: 14.59–20.60 Ma), corresponding to the transition from the early to middle Miocene.

## 3. Discussion

### 3.1. Comparative Plastome Features and Structural Evolution

Our study presents the first complete chloroplast genome of *E. scapiflorum*, a characteristic alpine species of the QTP. The assembled plastome is 153,041 bp in length and exhibits a characteristic quadripartite structure. Its gene organization, composition, and overall GC content (36.41%) are consistent with those reported for other species [18,19]. Although the observed variation in GC content across structural regions (IRs > LSC > SSC) is consistent with the known pattern driven by GC-rich rRNA genes in the IRs [20], this feature alone does not distinguish *E. scapiflorum* from its close relatives. The annotation identified 132 genes, including 87 protein-coding genes, which represents the conserved gene repertoire typical of autotrophic Brassicaceae plastomes [21].

A more informative finding emerges from comparative analysis of IR boundaries: unlike the pronounced expansion or contraction observed in some alpine or short-lived Brassicaceae species (e.g., significant IR expansion into single-copy regions in *C. tenella*; Figure 4), *E. scapiflorum* exhibits highly conserved IR/SC boundaries relative to closely related taxa. This structural stability is further supported by whole-genome synteny alignments, which reveal no large-scale inversions or rearrangements (Figure 3). Consequently, macrostructural plastome variation does not appear to be a primary evolutionary driver in this lineage. Nevertheless, the presence of dispersed repeats, particularly palindromic sequences, however, indicates potential recombination hotspots that could facilitate structural variation over extended evolutionary timescales [22,23].

### 3.2. Genome Divergence, Hotspots, and Implications for Molecular Markers

Our nucleotide diversity analysis identified several highly variable regions in *E. scapiflorum* and related taxa. Consistent with angiosperm-wide patterns [24], intergenic spacers and specific coding regions exhibited elevated Pi values. Notably, *accD* and *ycf1* were the most divergent protein-coding genes (Pi > 0.09). The *ycf1* gene, spanning the SSC/IR boundary, is widely recognized as one of the most variable plastid genes. It has proven valuable for resolving shallow phylogenetic relationships [24], a utility that can now be extended to *E. scapiflorum*.

Furthermore, SSR analysis revealed 78 loci, with >50% located in intergenic spacers. The prevalence of A/T-rich repeats in non-coding regions, which is common across flowering plants, contributes to their high polymorphism and makes them excellent candidates for population-specific microsatellite markers. Future studies should leverage these SSRs and hypervariable regions (e.g., *trnH-psbA*, *rpl32-trnL*) to investigate the genetic diversity, population structure, and phylogeography of *E. scapiflorum*. Such efforts, particularly across the topographically complex QTP, will be crucial for understanding the species’ response to Quaternary climatic oscillations [25].

### 3.3. Evolutionary Constraints and Positive Selection

Analysis of *Ka*/*Ks* substitution rates reveals that the majority of plastid genes in *E. scapiflorum* exhibit *Ka*/*Ks* ratios significantly below 1, consistent with purifying selection maintaining the integrity of genes critical for photosynthesis and metabolism [26]. However, seven genes (*ycf2*, *ndhE*, *cemA*, *clpP*, *psbH*, *ycf4*, *ndhB*) showed *Ka*/*Ks* > 1. While this pattern is statistically consistent with relaxed selective constraints or positive selection, we caution against overinterpreting these values as definitive evidence of adaptive evolution without additional statistical (e.g., branch-site models) or functional validation [27]. With this caveat, *clpP* (encoding a proteolytic subunit) and *psbH* (involved in photosystem II stabilization) emerge as plausible candidates for further investigation, given their known roles in protein homeostasis and photosynthetic efficiency under environmental stress [27,28]. Additionally, the *accD* gene, which is essential for fatty acid biosynthesis, exhibited the highest nucleotide diversity (Pi = 0.13031). Collectively, these genes warrant targeted study to test whether they contribute to molecular adaptation of *E. scapiflorum* to alpine abiotic stresses (e.g., intense UV radiation, freezing temperatures, oxidative stress). At present, the detected signals are best interpreted as hypothesis-generating rather than conclusive evidence of positive selection.

### 3.4. Phylogenetic Implications and Taxonomic Insights

Our phylogenomic analyses robustly resolve *A. petiolata* as the sister taxon to *E. scapiflorum.* This relationship illuminates a striking ecological divergence: *A. petiolata* is a biennial herb restricted to forest understories, whereas *E. scapiflorum* is a cushion-forming perennial specialized for high-altitude scree slopes (>3500 m). Given the conserved chloroplast genomic architecture in *E. scapiflorum*, this dramatic niche differentiation suggests that their common ancestor possessed substantial adaptive plasticity, enabling divergent responses to contrasting selective regimes [2,29]. We therefore interpret the cushion-forming perennial habit of *E. scapiflorum* as a derived character state reflecting specialized adaptation to persistent alpine stressors. This finding is consistent with the hypothesis of evolutionary lability and convergent evolution of life-history strategies within alpine Brassicaceae [30].

The estimated divergence time (ca. 17.57 Ma, 95% HPD: 14.59–20.60 Ma) places the evolutionary split of this lineage firmly within the early to middle Miocene. This timeframe, encompassing the estimated HPD interval, coincides temporally with major orogenic uplift and climatic cooling of the Plateau and Himalayas. These Neogene environmental shifts likely promoted diversification and adaptive evolution of the lineage, ultimately leading to the endemic establishment of *E. scapiflorum* [2,16,17,31]. Thus, its evolutionary trajectory appears closely linked to the formation of new alpine habitats and ecological niches during plateau orogenesis. Colonization of scree slope environments may represent a key adaptation that enabled the lineage to exploit these nascent niches, promoting its survival and diversification amidst profound geological heterogeneity.

We acknowledge that evolutionary inferences based solely on the plastid genome are subject to limitations due to its uniparental (maternal) inheritance. Future studies employing nuclear markers or biparentally inherited loci will be necessary to validate the divergence times and phylogenetic relationships presented here.

## 4. Materials and Methods

### 4.1. Experimental Materials

Fresh and healthy leaves of *E. scapiflorum* were collected in Maduo County, Qinghai Province (97°51′54″ E, 34°24′40″ N). The collected leaves were immediately dried and preserved using color-changing silica gel. The DNA voucher has been deposited at the College of Agriculture and Animal Husbandry, Qinghai University (voucher No. 2024-026). Collection was conducted with approval and in compliance with local biodiversity regulations.

### 4.2. Total DNA Extraction

Genomic DNA was extracted from *E. scapiflorum* leaf tissue using a modified CTAB (cetyltrimethylammonium bromide) method [32]. The integrity of the extracted DNA was subsequently assessed by electrophoresis on a 1.0% agarose gel. Its concentration and purity were then determined by measuring the OD_260/280_ ratio using a Nanodrop 2000 spectrophotometer (Thermo Fisher Scientific, Wilmington, DE, USA).

### 4.3. Sequencing, Assembly, and Annotation

The quality-controlled DNA samples were randomly fragmented using a Covaris ultrasonicator (Covaris LLC, Woburn, MA, USA). The DNA fragments underwent end repair, poly-A tailing at the 3′ end, adapter ligation, and purification. Then, a sequencing library was constructed with PCR amplification. Sequencing was performed on an Illumina NovaSeq 6000 platform (Genepioneer, Nanjing, China) to generate raw data. Raw sequencing reads were filtered using Fastp v0.23.2 [33] with default parameters to remove adapter sequences, low-quality reads (quality score < 20), short reads (<50 bp), and PCR duplicates. The complete chloroplast genome was de novo assembled using GetOrganelle v1.7.7 [34] with the embryophyte plastid database (-F embplant_pt), a k-mer gradient of 21, 45, 65, 85, and 105, and 16 threads.

Furthermore, the integrity of the inverted repeat (IR) boundaries was validated by mapping continuous reads across the junctions in Geneious v2023.0.1. The assembly graph and genome circularity were visually evaluated using Bandage v0.9.0. To validate the assembly and confirm circularization, approximately 5 Gb of raw reads were processed, filtered, and then remapped to the assembled plastome. After filtering and alignment, 3,275,427 paired-end reads were successfully mapped to the chloroplast genome, achieving an average coverage depth of 6797×. Initial annotation was annotated using the online tool GeSeq v2.03 (https://chlorobox.mpimp-golm.mpg.de/geseq.html, accessed on 10 September 2025) [35] (BLAT identity ≥ 85%), with the chloroplast genome of *Alliaria petiolata* (NC_049586.1) as a reference.

Subsequently, annotation was performed using the “2544-plastomes” database within the CPGAVAS2 software (http://47.96.249.172:16019/analyzer/home, accessed on 10 September 2025) [36]. Conflicting annotations between the two methods were resolved by manual curation based on sequence homology, open reading frame (ORF) integrity, and conserved gene structures in Brassicaceae. Finally, a circular map of the *E. scapiflorum* chloroplast genome was generated using OGDRAW v1.3.1 (OrganellarGenomeDRAW, https://chlorobox.mpimp-golm.mpg.de/OGDraw.html, accessed on 10 September 2025) [37].

### 4.4. Analysis of Chloroplast Genome Features and Repeat Sequences

Geneious Prime 2023.0.1 software [38] was employed to determine the total length of the *E. scapiflorum* chloroplast genome and the lengths of its structural regions, including the large single-copy (LSC), small single-copy (SSC), and inverted repeat (IR) regions. The types, numbers, and base composition of genes were also analyzed and compared to elucidate the sequence characteristics of the *E. scapiflorum* chloroplast genome. Simple sequence repeat (SSR) loci within the *E. scapiflorum* chloroplast genome sequence were identified using the online tool MISA (https://webblast.ipk-gatersleben.de/misa/index.php?action=1, accessed on 12 September 2025) [39,40]. The detection parameters for mono- to hexanucleotide repeats were set to 10, 6, 5, 3, 3, and 3, respectively. Dispersed repeat sequences in the *E. scapiflorum* chloroplast genome, including palindromic (P), complementary (C), forward (F), and reverse (R) repeats, were detected using the REPuter (https://bibiserv.cebitec.uni-bielefeld.de/reputer, accessed on 12 September 2025) [41]. The search parameters were set to a minimum repeat length of 30 bp, sequence similarity greater than 90%, and a Hamming distance of 3.

### 4.5. Chloroplast Genome Synteny Comparison

To identify structural rearrangements and inversions, collinearity analysis of the chloroplast genomes from eight species (including *E. scapiflorum*) was conducted using the progressiveMauve algorithm (Mauve v2.4.0) [42] within Geneious v2023.0.1, applying default parameters. The analysis was performed under default parameters, with the *E. scapiflorum* chloroplast genome serving as the reference.

The IRscope online tool (https://irscope.shinyapps.io/irapp/, accessed on 14 September 2025) [43] was employed to compare the inverted repeat (IR) region boundaries and analyze IR contraction and expansion among the eight chloroplast genome sequences, thereby detecting boundary shifts in the four genomic regions. The annotated chloroplast genome sequences were sequentially uploaded to automatically generate a visual comparative map. This was followed by manual verification of the positions of each region to ensure the accuracy of the comparison.

### 4.6. Nucleotide Diversity Calculation

The nucleotide polymorphism (Pi) value was calculated for the *E. scapiflorum* chloroplast genome using DnaSP v6.034 software [44] to identify regions of high nucleotide polymorphism in both coding and non-coding regions. The analysis was performed with a window length of 200 bp and a step size of 600 bp. The generated Pi value box plot was compared against the genome visualization map to determine the corresponding gene regions, and each variable region was annotated accordingly.

### 4.7. Ka/Ks Analysis

Nucleotide and amino acid sequences of protein-coding genes were extracted from the chloroplast genomes of *E. scapiflorum* and seven related species using PhyloSuite v1.2.2 [45]. Following the identification of homologous genes, nucleotide and amino acid sequences were aligned using ParaAT v2.0 [46] to produce .axt format files. Subsequently, the nonsynonymous (*K*a) and synonymous (*K*s) substitution rates were calculated using *K*a*k*s_Calculator 3.0 [47] for the eight species. The *K*a/*K*s ratio was used to evaluate selective pressure, where a ratio > 1 indicates positive selection and a ratio < 1 indicates purifying selection [48]. In the study, the genetic code table was set to 11 (Bacterial and Plant Plastid Code), and the GY calculation method (maximum-likelihood) was selected for analysis.

### 4.8. Phylogenetic Analysis

To further resolve the phylogenetic position of *E. scapiflorum* within the family, a more comprehensive dataset was constructed. Chloroplast genome sequences of 35 species representing various tribes within Brassicaceae were retrieved from the NCBI database (Table 5). Protein-coding sequences (CDS) were extracted from the chloroplast genomes of all 36 analyzed species using Geneious v2023.0.1. Multiple sequence alignments for both whole chloroplast genomes and the concatenated CDSs were performed using MAFFT v7.526 [49] integrated into PhyloSuite platform. The aligned sequences were subsequently trimmed using trimAl v1.5.0 [50]. Phylogenetic analyses were conducted using both Maximum Likelihood (ML) and Bayesian Inference (BI) methods.

The ML tree was constructed using IQ-TREE v2.2.0 [51] with codon partitioning, substitution models selected by ModelFinder v2.2.0, and 5000 ultrafast bootstrap replicates. The BI tree was inferred using MrBayes v3.2.7 [52] with two independent runs of four Markov chains for 10,000,000 generations, sampling every 1000 generations, and discarding the first 25% as burn-in. Convergence was confirmed in Tracer v1.7.1 [53] (effective sample size [ESS] > 200). Support values are presented as ML bootstrap/BI posterior probability at each node.

### 4.9. Divergence Time Estimation

Divergence times among the analyzed species were estimated based on the concatenated CDS using a Bayesian relaxed-clock model implemented in BEAST v2.7.3. Secondary calibration points were obtained from the TimeTree database (http://www.timetree.org/, accessed on 18 September 2025) [54] to constrain well-supported nodes. Specifically, the divergence times between *Nasturtium officinale* and *Armoracia rusticana* (7.4 Ma, 95% HPD: 6.3–8.5 Ma), and between *Barbarea vulgaris* and *Rorippa sylvestris* (6.4 Ma, 95% HPD: 3.3–14.0 Ma) were employed as calibration constraints. A normal prior distribution was applied to these secondary calibrations, as it symmetrically accommodates the mean ages and their empirical 95% confidence intervals derived from the TimeTree database, minimizing subjective parameterization. The analysis utilized a Yule speciation prior, which is appropriate for species-level phylogenies. Given the strong phylogenetic signal provided by the concatenated CDS dataset, the posterior estimates are expected to be robust and primarily driven by the sequence data rather than prior sensitivity. The Markov Chain Monte Carlo (MCMC) was run for 100 million generations, sampling every 1000 generations. Convergence was evaluated in Tracer v1.7.1, ensuring all parameters reached an ESS > 200. The final maximum clade credibility tree was generated using TreeAnnotator v2.7.8 with a 10% burn-in, and node ages were reported with 95% highest posterior density (HPD) intervals to represent uncertainty.

## 5. Conclusions

This study reported the first complete chloroplast genome of *E. scapiflorum*, a characteristic alpine species endemic to the QTP. Our analyses resolved its precise phylogenetic position, revealed a highly conserved plastome structure, and identified candidate genes potentially under positive selection associated with high-altitude adaptation. The divergence time estimation linked its speciation of *E. scapiflorum* to the dynamic geological history of the QTP. The comprehensive genomic data generated herein provide a foundational resource for future evolutionary and ecological studies. Specifically, the identified SSRs, particularly those located in hypervariable intergenic spacer regions, provided valuable resources for population genetic studies of *E. scapiflorum*, enabling the exploration of its genetic structure, demographic history, and local adaptation. Future research should focus on expanding sampling across the species’ distribution to test phylogeographic hypotheses using these markers. Furthermore, integrating nuclear genomic data and conducting functional studies of positively selected genes (e.g., *psbH*, *clpP*) will be crucial to validate their role in alpine adaptation and to fully unravel the genomic basis of survival in one of Earth’s most extreme environments.

## Figures and Tables

**Table 1:** Gene composition in the chloroplast genome of *E. scapiflorum*.

Category	Gene Group	Gene Name
Photosynthesis	Subunits of photosystem I	*psaA*, *psaB*, *psaC*, *psaI*, *psaJ*
	Subunits of photosystem II	*psbA*, *psbB*, *psbC*, *psbD*, *psbE*, *psbF*, *psbH*, *psbI*, *psbJ*, *psbK*, *psbL*, *psbM*, *psbN*, *psbT*, *psbZ*
	Subunits of NADH dehydrogenase	ndhA*, ndhB*(2), *ndhC*, *ndhD*, *ndhE*, *ndhF*, *ndhG*, *ndhH*, *ndhI*, *ndhJ*, *ndhK*
	Subunits of cytochrome b/f complex	*petA*, petB*, petD*, *petG*, *petL*, *petN*
	Subunits of ATP synthase	*atpA*, *atpB*, *atpE*, atpF*, *atpH*, *atpI*
	Large subunit of rubisco	*rbcL*
Self-replication	Proteins of large ribosomal subunit	*rpl14*, rpl16*, rpl2*(2), *rpl20*, *rpl22*, *rpl23(2)*, *rpl32*, *rpl33*, *rpl36*
	Proteins of small ribosomal subunit	*rps11*, rps12**(2), *rps14*, *rps15*, rps16*, *rps18*, *rps19*, *rps2*, *rps3*, *rps4*, *rps7(2)*, *rps8*
	Subunits of RNA polymerase	*rpoA*, *rpoB*, rpoC1*, *rpoC2*
	Ribosomal RNAs	*rrn16(2)*, *rrn23(2)*, *rrn4.5(2)*, *rrn5(2)*
	Transfer RNAs	trnA-UGC*(2), *trnC-GCA*, *trnD-GUC*, *trnE-UUC*, *trnF-GAA*, trnG*, *trnG-UCC*, *trnH-GUG*, *trnI-CAU(2)*, trnI-GAU*(2), trnK-UUU*, *trnL-CAA(2)*, trnL-UAA*, *trnL-UAG*, *trnM-CAU*, *trnN-GUU(2)*, *trnP-UGG*, *trnQ-UUG*, *trnR-ACG(2)*, *trnR-UCU*, *trnS-GCU*, *trnS-GGA*, *trnS-UGA*, *trnT-GGU*, *trnT-UGU*, *trnV-GAC(2)*, trnV-UAC*, *trnW-CCA*, *trnY-GUA*, *trnfM-CAU*
Other genes	Maturase	*matK*
	Protease	clpP**
	Envelope membrane protein	*cemA*
	Acetyl-CoA carboxylase	*accD*
	c-type cytochrome synthesis gene	*ccsA*
Genes of unknown function	Conserved hypothetical chloroplast ORF	#*ycf1*, *ycf1*, *ycf15(2)*, *ycf2(2)*, ycf3**, *ycf4*

Notes: Gene*: Gene with one introns; Gene**: Gene with two introns; #Gene: Pseudo gene; Gene(2): Number of copies of multi-copy genes.

**Table 2:** Reactors and numbers of SSRs in the chloroplast genome of *E. scapiflorum*.

Region	Exon	Intron	Intergenic
LSC	37	31	129
SSC	40	4	19
IR	22	4	16
	32.78%	12.91%	54.30%

**Table 3:** Statistics of the analysis results of scattered repetitive sequences.

Length of Repeat (bp)	Forward	Palindromic	Reverse	Complement	Total
30	5	7	0	1	13
31	2	0	0	0	2
32	2	1	0	0	3
33	2	2	0	0	4
34	0	0	1	0	1
36	0	1	0	0	1
37	1	0	0	0	1
39	0	1	0	0	1
41	0	1	0	0	1
43	1	0	0	0	1
44	0	2	0	0	2
46	1	1	0	0	2
55	0	1	0	0	1

**Table 4:** Comparison of IR/SC boundary positions (bp) among eight Brassicaceae chloroplast genomes.

Species	LSC Length	IRa Length	SSC Length	IRb Length	Total Length	JLB	JSB	JSA	JLA
*E. scapiflorum*	83,547	25,994	17,506	25,994	153,041	*ycf2*	*ycf1*	*ycf1*	*ycf2*
*A. petiolata*	82,927	26,454	18,014	26,454	153,849	*ycf2*	*ycf1*	*ycf1*	*ycf2*
*C. himalaica*	83,421	26,378	17,764	26,378	153,941	*ycf2*	*ycf1*	*ycf1*	*ycf2*
*S. cheiranthoides*	83,672	26,295	17,643	26,295	153,905	*ycf2*	*ycf1*	*ycf1*	*ycf2*
*T. glabra*	83,714	26,183	17,826	26,183	153,906	*ycf2*	*ycf1*	*ycf1*	*ycf2*
*C. limprichtiana*	84,104	26,077	17,419	26,077	153,677	*ycf2*	*ycf1*	*ycf1*	*ycf2*
*L. apetalum*	84,362	26,245	17,531	26,245	154,383	*ycf2*	*ycf1*	*ycf1*	*ycf2*
*C. tenella*	85,364	29,399	12,504	29,399	156,666	*rps19*	*ycf1*	*ycf1*	*rps19*

Note: JLB: junction between IRb and LSC; JSB: junction between IRb and SSC; JSA: junction between SSC and IRa; JLA: junction between IRa and LSC. Boundary positions are given as gene names located at each junction.

**Table 5:** Information table of tested species.

Tribe	Genus	Species	GenBank ID
Arabideae	*Arabidopsis*	*Arabidopsis halleri*	NC_034366.1
		*Arabidopsis lyrata*	NC_034365.1
	*Arabis*	*Arabis flagellosa*	NC_037475.1
		*Arabis hirsuta*	NC_009268.1
		*Arabis paniculata*	NC_053754.1
	*Barbarea*	*Barbarea vulgaris*	NC_081109.1
	*Christolea*	*Christolea crassifolia*	NC_050790.1
	*Dendroarabis*	*Dendroarabis fruticulosa*	NC_062040.1
	*Dontostemon*	*Dontostemon elegans*	NC_081114.1
		*Dontostemon micranthus*	NC_049628.1
	*Nasturtium*	*Nasturtium officinale*	NC_009275.1
	*Rorippa*	*Rorippa globosa*	NC_070416.1
		*Rorippa sylvestris*	NC_069649.1
	*Stevenia*	*Stevenia cheiranthoides*	MK_637795.1
	*Turritis*	*Turritis glabra*	NC_061285.1
Brassiceae	*Brassica*	*Brassica oleracea*	NC_041167.1
	*Cardamine*	*Cardamine limprichtiana*	NC_034287.1
	*Raphanus*	*Raphanus sativus*	NC_024469.1
	*Orychophragmus*	*Orychophragmus violaceus*	NC_049687.1
Conringieae	*Conringia*	*Conringia planisiliqua*	NC_049619.1
Drabeae	*Armoracia*	*Armoracia rusticana*	NC_060501.1
Euclidieae	*Euclidium*	*Euclidium syriacum*	NC_081125.1
Hesperideae	*Cryptospora*	*Cryptospora falcata*	NC_050791.1
Lepidieae	*Capsella*	*Capsella bursa-pastoris*	NC_009270.1
	*Iberis*	*Iberis amara*	NC_049655.1
	*Isatis*	*Isatis gymnocarpa*	NC_081128.1
	*Lepidium*	*Lepidium apetalum*	NC_051540.1
	*Pugionium*	*Pugionium cornutum*	NC_030516.1
Matthioleae	*Solms-laubachia*	*Solms-laubachia eurycarpa*	NC_034359.1
		*Solms-laubachia lanata*	NC_050812.1
		*Solms-laubachia mieheorum*	NC_053342.1
		*Solms-laubachia platycarpa*	NC_050816.1
Sisymbrieae	*Alliaria*	*Alliaria petiolata*	NC_062040.1
	*Crucihimalaya*	*Crucihimalaya himalaica*	NC_061290.1
	*Eutrema*	*Eutrema scapiflorum* *	PX_526263.1
	*Sisymbrium*	*Sisymbrium altissimum*	NC_059802.1

Note: * The newly assembled chloroplast genome sequence was used in this study.

## Data Availability

The chloroplast genomes of *E. scapiflorum* under study are deposited in the GenBank database under the following accession numbers: PX526263.1. The other sequences used in this study were downloaded from the NCBI.

## Supplementary Materials

The following supporting information can be downloaded at: https://www.mdpi.com/article/10.3390/ijms27125195/s1.

## Abbreviations

cp: Chloroplast; QTP: Qinghai–Tibet Plateau; LSC: Large Single-Copy region; SSC: Small Single-Copy region; IR: Inverted repeat; tRNA: Transfer RNA; rRNA: Ribosomal RNA; SSR: Simple sequence repeat; Ka: Nonsynonymous Substitution Rate; Ks: Synonymous Substitution Rate; Ma: Mega-annum; ITS: Internal Transcribed Spacer; CTAB: Cetyltrimethylammonium bromide; OD: Optical Density; PCR: Polymerase Chain Reaction; P: Palindromic; C: Complementary; F: Forward; R: Reverse; Pi: Nucleotide Polymorphism; CDS: Protein-coding Sequence; ML: Maximum Likelihood; BI: Bayesian Inference; 95% HPD: 95% Highest Posterior Density; MCMC: Markov Chain Monte Carlo; ORF: Open Reading Frame; IGS: Intergenic Spacer; LCBs: Locally Collinear Blocks; SNP: Single Nucleotide Polymorphism; BS: Bootstrap Support; PP: Posterior Probability; UV: Ultraviolet Light.
