# Supplementary material for: Chloroplast Phylogenomics and Evolutionary History of the Alpine Endemic Eutrema scapiflorum
Source: Int J Mol Sci. 2026 Jun 8;27(12):5195. doi: 10.3390/ijms27125195 (PMC13299512; doi:10.3390/ijms27125195)
Supplement: Supplementary file 1 [file ijms-27-05195-s001.zip › ijms-4246667-supplementary.pdf]

Table S1 The Pi Value Statistics

No.	Region	Gene name	Pi Value	Total Number of mutations	Region length
1	IR	*trnN-GUU*	0.02778	8	74
2	IR	*rps7*	0.00527	8	468
3	IR	*ndhB*	0.00491	16	1,539
4	IR	*trnL-CAA*	0.00838	2	83
5	IR	*ycf15*	0.02599	9	234
6	IR	*ycf2*	0.02794	600	7,326
7	IR	*trnI-CAU*	0	0	75
8	IR	*rpl23*	0.01342	14	282
9	IR	*rpl2*	0.00905	26	828
10	IR	*trnR-ACG*	0.01255	3	75
11	IR	*rrn5*	0	0	121
12	IR	*rrn4.5*	0.00243	1	103
13	IR	*rrn23*	0.00306	30	2,810
14	IR	*trnA-UGC*	0.00685	2	73
15	IR	*trnI-GAU*	0.01389	4	77
16	IR	*rrn16*	0.00304	17	1,491
17	IR	*trnV-GAC*	0	0	72
18	LSC	*trnH-GUG*	0	0	74
19	LSC	*trnG*	0.01761	5	77
20	LSC	*trnR-UCU*	0.01042	3	72
21	LSC	*atpA*	0.0263	129	1,524
22	LSC	*atpF*	0.03391	58	555
23	LSC	*atpH*	0.01728	15	246
24	LSC	*atpI*	0.02167	48	750
25	LSC	*rps2*	0.0224	53	711
26	LSC	*rpoC2*	0.03421	434	4,146
27	LSC	*rpoC1*	0.01954	133	2,043
28	LSC	*rpoB*	0.02218	226	3,219
29	LSC	*psbA*	0.01594	51	1,062
30	LSC	*trnC-GCA*	0.00704	2	79
31	LSC	*petN*	0.01706	5	90
32	LSC	*psbM*	0.03401	8	105
33	LSC	*trnD-GUC*	0.01272	3	78
34	LSC	*trnY-GUA*	0.0051	1	86
35	LSC	*trnE-UUC*	0.01637	4	75
36	LSC	*trnT-GGU*	0	0	74
37	LSC	*psbD*	0.01359	47	1,062
38	LSC	*psbC*	0.01518	72	1,422
39	LSC	*trnS-UGA*	0	0	93
40	LSC	*trnK-UUU*	0.00347	1	73

41	LSC	*psbZ*	0.00926	7	189
42	LSC	*trnG-UCC*	0.01459	3	72
43	LSC	*trnfM-CAU*	0.014	3	75
44	LSC	*rps14*	0.01968	19	303
45	LSC	*psaB*	0.01579	113	2,205
46	LSC	*psaA*	0.014	105	2,253
47	LSC	*ycf3*	0.01097	13	507
48	LSC	*trnS-GGA*	0.00616	1	89
49	LSC	*rps4*	0.02157	42	606
50	LSC	*trnT-UGU*	0.00342	1	75
51	LSC	*matK*	0.04418	222	1,593
52	LSC	*trnL-UAA*	0.00924	2	87
53	LSC	*trnF-GAA*	0.00783	1	73
54	LSC	*ndhJ*	0.02646	38	477
55	LSC	*ndhK*	0.019	41	678
56	LSC	*ndhC*	0.02706	29	363
57	LSC	*trnV-UAC*	0.00676	2	76
58	LSC	*trnM-CAU*	0	0	73
59	LSC	*atpE*	0.02184	30	399
60	LSC	*atpB*	0.02498	125	1,509
61	LSC	*rbcL*	0.01674	73	1,449
62	LSC	*rps16*	0.04377	24	268
63	LSC	*accD*	0.13031	607	2,165
64	LSC	*psaI*	0.02381	9	114
65	LSC	*ycf4*	0.0455	87	687
66	LSC	*cemA*	0.02505	60	702
67	LSC	*petA*	0.02095	62	963
68	LSC	*psbJ*	0.01316	5	123
69	LSC	*psbL*	0.01526	5	117
70	LSC	*psbF*	0.01131	4	120
71	LSC	*psbE*	0.00879	8	252
72	LSC	*petL*	0.01525	4	96
73	LSC	*trnQ-UUG*	0.00347	1	72
74	LSC	*petG*	0.01974	6	114
75	LSC	*trnW-CCA*	0.00342	1	74
76	LSC	*trnP-UGG*	0	0	76
77	LSC	*psaJ*	0.02962	10	141
78	LSC	*rpl33*	0.02061	12	201
79	LSC	*rps18*	0.02007	22	306
80	LSC	*rpl20*	0.04136	48	354
81	LSC	*rps12*	0.0073	10	372
82	LSC	*clpP*	0.05729	127	603

83	LSC	*psbB*	0.02365	111	1,527
84	LSC	*psbK*	0.01728	10	186
85	LSC	*psbT*	0.02311	8	102
86	LSC	*psbN*	0.00568	3	132
87	LSC	*psbH*	0.03169	24	222
88	LSC	*petB*	0.01257	28	648
89	LSC	*petD*	0.0173	25	483
90	LSC	*rpoA*	0.02127	69	996
91	LSC	*rps11*	0.02372	34	417
92	LSC	*rpl36*	0.03102	12	114
93	LSC	*rps8*	0.03245	41	405
94	LSC	*rpl14*	0.02458	29	369
95	LSC	*psbI*	0.01126	5	111
96	LSC	*rpl16*	0.02232	28	408
97	LSC	*rps3*	0.02957	61	657
98	LSC	*rpl22*	0.04747	69	483
99	LSC	*rps19*	0.02471	22	279
100	LSC	*trnS-GCU*	0.01136	3	88
101	SSC	*ndhF*	0.04175	272	2,256
102	SSC	*ndhA*	0.02986	69	1,083
103	SSC	*ndhH*	0.02802	33	1,182
104	SSC	*rps15*	0.03304	30	267
105	SSC	*ycf1*	0.09711	1,359	5,775
106	SSC	*rpl32*	0.02875	14	174
107	SSC	*trnL-UAG*	0.01518	3	82
108	SSC	*ccsA*	0.0482	148	999
109	SSC	*ndhD*	0.03213	135	1,575
110	SSC	*psaC*	0.02192	17	246
111	SSC	*ndhE*	0.02365	25	307
112	SSC	*ndhG*	0.03132	51	531
113	SSC	*ndhI*	0.02976	41	545
